# In Vivo Validation of Restored Chordal Biomechanics After Mitral Ring Annuloplasty in a Rare Ovine Case of Natural Chronic Functional Mitral Regurgitation

**DOI:** 10.3390/jcdd7020017

**Published:** 2020-05-15

**Authors:** Hanjay Wang, Michael J. Paulsen, Annabel M. Imbrie-Moore, Yuko Tada, Hunter Bergamasco, Sam W. Baker, Yasuhiro Shudo, Michael Ma, Y. Joseph Woo

**Affiliations:** 1 Department of Cardiothoracic Surgery, Stanford University, Stanford, CA 94305, USA; hanjay@stanford.edu (H.W.); mpaulsen@stanford.edu (M.J.P.); aimbrie@stanford.edu (A.M.I.-M.); hbergamasco@stanfordhealthcare.org (H.B.); yshudo@stanford.edu (Y.S.); mma@stanford.edu (M.M.); 2 Stanford Cardiovascular Institute, Stanford University, Stanford, CA 94305, USA; ytada@stanford.edu; 3 Department of Mechanical Engineering, Stanford University, Stanford, CA 94305, USA; 4 Department of Cardiovascular Medicine, Stanford University, Stanford, CA 94305, USA; 5 Department of Comparative Medicine, Stanford University, Stanford, CA 94305, USA; sambaker@stanford.edu; 6 Department of Bioengineering, Stanford University, Stanford, CA 94305, USA

**Keywords:** mitral valve, regurgitation, annuloplasty, biomechanics, chordae tendineae

## Abstract

Mitral valve chordae tendineae forces are elevated in the setting of mitral regurgitation (MR). Ring annuloplasty is an essential component of surgical repair for MR, but whether chordal forces are reduced after mitral annuloplasty has never been validated in vivo. Here, we present an extremely rare ovine case of natural, severe chronic functional MR, in which we used force-sensing fiber Bragg grating neochordae to directly measure chordal forces in the baseline setting of severe MR, as well as after successful mitral ring annuloplasty repair. Overall, our report is the first to confirm in vivo that mitral ring annuloplasty reduces elevated chordae tendineae forces associated with chronic functional MR.

## 1. Introduction

Ring annuloplasty is an essential component of mitral valve repair for the correction of mitral regurgitation (MR). Previous ex vivo studies have demonstrated that chordae tendineae forces are significantly elevated under simulated conditions of functional MR due to annular dilation and loss of leaflet coaptation [1] and that mitral annuloplasty may reduce these elevated chordal forces [2]. Because chordal force distribution impacts valve performance [3], the extent to which chordal biomechanics are recovered postoperatively may affect the long-term durability and outcomes of mitral repair. However, the fundamental concept that chordal forces are reduced after mitral annuloplasty has not been validated in vivo, due to the absence of large animal models with natural chronic MR and the ethical impermissibility of performing biomechanics experiments in patients. Here, we present an extremely rare ovine case of natural, severe chronic functional MR, in which we tested the hypothesis that ring annuloplasty repair of functional MR reduces chordae tendineae forces.

## 2. Case Report

**Figure 1:**
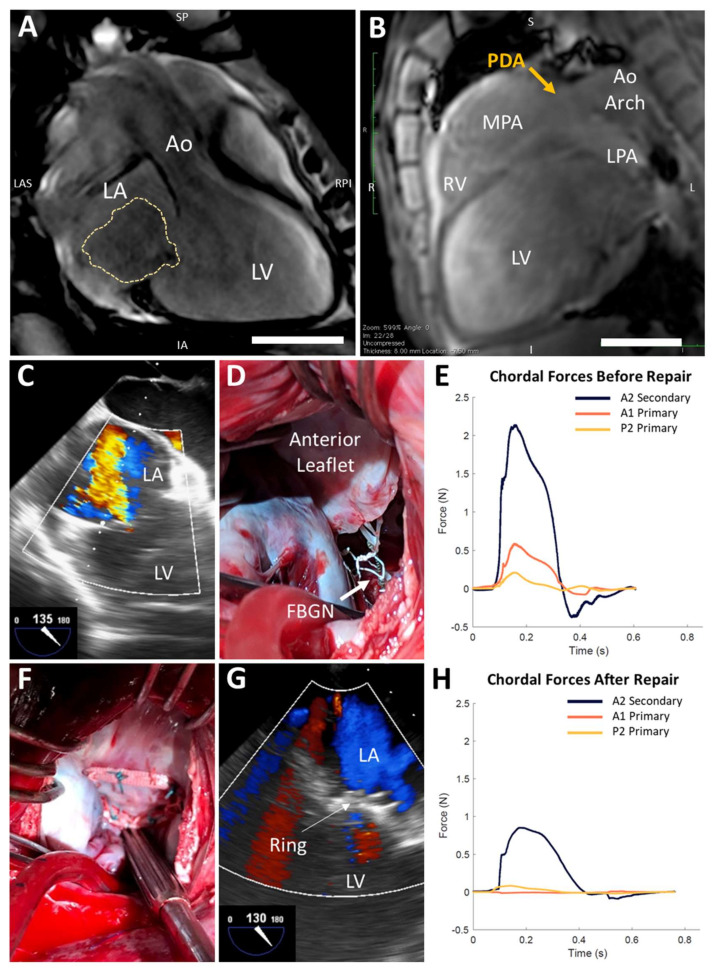
Chordal forces are reduced after mitral ring annuloplasty in a rare ovine case of natural severe functional mitral regurgitation. (**A**,**B**) Cardiac magnetic resonance imaging reveals left atrial (LA) and left ventricular (LV) dilation, central mitral regurgitation (MR, yellow trace), and a patent ductus arteriosus (PDA). Aorta (Ao); inferior (I); inferior-anterior (IA); left (L); left anterior-superior (LAS); left pulmonary artery (LPA); main pulmonary artery (MPA); right (R); right posterior-inferior (RPI); right ventricle (RV); superior (S); superior-posterior (SP). Scale bars, 5 cm. (**C**) Baseline transesophageal echocardiography (TEE) confirms severe MR. (**D**) Instrumentation of native mitral valve chordae with force-sensing fiber Bragg grating neochordae (FBGN, white arrow). (**E**) Chordal forces in the state of severe MR over a representative cardiac cycle. (**F**) Mitral ring annuloplasty repair. (**G**) TEE confirms no MR after annuloplasty ring implantation. (**H**) Chordal forces are reduced after annuloplasty ring implantation.

A male Dorset sheep (6 months old, 40 kg) was diagnosed on cardiac magnetic resonance imaging (MRI) with left atrial (LA), left ventricular (LV), and mitral annular dilation (Table 1), as well as prominent central MR (Figure 1A). The sheep was planned for a terminal procedure involving mitral annuloplasty repair on cardiopulmonary bypass (CPB) with biomechanical analysis of chordal forces. The experiment was performed in accordance with the United States National Institutes of Health “Guide for the Care and Use of Laboratory Animals” (8th Edition, 2011), with approval by the Institutional Animal Care and Use Committee at Stanford University. Our strategy for sedation, anesthesia, analgesia, anticoagulation, and cardioplegia in ovine cardiac surgery involving CPB was described previously [4].

The sheep was intubated, and a right thoracotomy was performed. A large 1.5 cm patent ductus arteriosus (PDA) was discovered, suggesting over-circulation as the etiology of LV dilation and functional MR. Retrospectively, the PDA could be identified on MRI, although dedicated sequences were not obtained (Figure 1B). The PDA was ligated and divided. Baseline transesophageal echocardiography (TEE) confirmed severe holosystolic MR (jet area 40.4% of total LA area, vena contracta 8.6 mm, Figure 1C). CPB was initiated via central aortic and bicaval cannulation. The heart was arrested with Del Nido cardioplegia, and a left atriotomy was made. Inspection of the mitral valve revealed a dilated annulus with intact subvalvular apparatus.

One secondary chord to the anterior leaflet (AS) and two primary chordae, one each to the anterior (AP) and posterior leaflets (PP), were instrumented with custom-designed force-sensing fiber Bragg grating neochordae (FBGN) [5]. For each target chord, a long 14 G needle was inserted into the papillary muscle tip along the same vector as the chord, exiting through the LV apex and left chest. The FBGN was passed retrograde through the needle into the LV and secured at its two ends to the target chord using 5–0 polytetrafluoroethylene sutures. The chord was cut between the sutures, transferring the load to the FBGN (Figure 1D). The LA was closed, the aortic clamp was removed, the heart resumed a normal rhythm, and CPB was weaned off. TEE confirmed no change in severe MR after FBGN placement (jet area 40.9%, vena contracta 7.7 mm). With stable hemodynamics off CPB (heart rate 80–100 beats/min, mean arterial pressure 65–70 mmHg, central venous pressure 4–6 mmHg), chordal forces were measured in the state of severe MR (Figure 1E). Peak forces in the AS, AP, and PP chordae were 2.14 N, 0.59 N, and 0.21 N, respectively. Systolic dF/dt (i.e., the time derivative of force) for the AS, AP, and PP chordae were 1441.8 N/s, 969.9 N/s, and 550.5 N/s, respectively.

Next, the heart was re-arrested on CPB and the mitral valve was repaired with a 28 mm Carpentier–Edwards Physio II ring (Edwards Lifesciences, Irvine, CA) (Figure 1F). Cross-clamp and CPB times for the entire procedure were 150 and 261 min, respectively. Post-repair TEE confirmed no MR (Figure 1G). Controlling for the same hemodynamics above, post-repair peak forces in the AS, AP, and PP chordae were 0.85 N, 0.01 N, and 0.08 N, respectively (Figure 1H). Systolic dF/dt for the AS, AP, and PP chordae were 54.2 N/s, 1.4 N/s, and 3.8 N/s, respectively. At the conclusion of the experiment, the sheep was euthanized via intravenous potassium chloride.

## 3. Discussion

Our report is the first to validate in vivo that mitral ring annuloplasty reduces elevated chordal forces associated with chronic functional MR. The in vivo forces we measured pre- and post-repair agreed well with previous ex vivo data assessing the impact of progressive annular dilation on chordal biomechanics [1].

Our unique ovine case involved severe chronic MR that developed naturally. Chronic left-to-right shunting through a large PDA resulted in persistent left heart volume overload, leading to gradual LA and LV dilation [6] and ultimately culminating in mitral annular dilation and development of functional MR. This pathophysiologic mechanism of functional MR has also been observed in human patients with PDA [7,8].

Although we draw inferences from only one animal, due to its extreme rarity, ex vivo experiments are currently unable to precisely replicate the motion of the mitral annulus, papillary muscles, and left ventricle wall, all of which directly influence valvular biomechanics. Furthermore, chordal forces cannot be safely analyzed in vivo in human patients with mitral valve disease, and no reproducible in vivo large animal model of natural MR exists. Indeed, to our knowledge, a PDA has never previously been reported to result in severe MR in a sheep [9]. Although induced chronic MR models caused by chord cutting [10], LV-to-LA shunt creation [11], or myocardial infarction are available [12], our rare sheep with natural, severe functional MR precisely mirrors a clinical patient referral without prior manipulation of the mitral valve apparatus or LV, and therefore represents the most translationally-optimal opportunity to study the biomechanical foundation upon which mitral annuloplasty repair is based.

The use of FBGN in an in vivo setting carries some inherent limitations. For example, it is possible that, in the course of replacing the native chordae with FBGN sensors, we may have inadvertently altered the MR disease state from the baseline. However, TEE imaging provided quantitative evidence of persistently severe central MR after FBGN implantation. In addition, the forces we measured in the in vivo state of severe, chronic functional MR were relatively similar to those observed in a previous ex vivo MR model [1]. Furthermore, elimination of MR in vivo by ring annuloplasty repair resulted in a reduction of chordal forces to a level similar to that observed in a previous ex vivo mitral repair model [13]. These results of our in vivo experiment therefore support and build upon the existing ex vivo data describing mitral valve biomechanics.

Overall, we report the first in vivo biomechanical validation that mitral ring annuloplasty reduces abnormally elevated chordae tendineae forces associated with chronic functional MR. Future long-term studies including sheep with induced MR versus healthy controls are required to confirm our observations.

## Figures and Tables

**Table 1:** Left heart measurements by cardiac magnetic resonance imaging.

Left Heart Measurements	MR Sheep	Healthy Sheep
Left Atrium Diameter	5.26 cm	2.83 ± 0.24 cm
Left Atrium Volume	229.0 mL	31.5 ± 8.2 mL
Left Ventricle Diameter	7.94 cm	4.49 ± 0.30 cm
Left Ventricle Volume	368.9 mL	103.6 ± 17.9 mL
Mitral Annulus Diameter	5.57 cm	3.08 ± 0.07 cm
Mitral Annulus Area	24.3 cm^2^	6.7 ± 1.5 cm^2^

Measurements were recorded at end-diastole for a rare male sheep with natural severe functional mitral regurgitation (MR) and compared to three representative healthy male sheep with the same age and weight (data presented as mean ± standard deviation). Diameter measurements are presented as the average diameters from the 2-chamber and 4-chamber views on cardiac magnetic resonance imaging.
